# Cumulative smoking dose affects the clinical outcomes of EGFR-mutated lung adenocarcinoma patients treated with EGFR-TKIs: a retrospective study

**DOI:** 10.1186/s12885-018-4691-0

**Published:** 2018-07-28

**Authors:** In Ae Kim, Jong Sik Lee, Hee Joung Kim, Wan Seop Kim, Kye Young Lee

**Affiliations:** 1 Lung Cancer Center, Konkuk University Medical Center, 120-1 Hwayang-dong, Gwangjin-Gu, Seoul, 05030 Republic of Korea; 2 Department of Pulmonary Medicine, Konkuk University School of Medicine, 120-1 Hwayang-dong, Gwangjin-Gu, Seoul, 05030 Republic of Korea; 3 Department of Pathology, Konkuk University School of Medicine, Seoul, Republic of Korea

**Keywords:** Cumulative smoking dose, EGFR mutations, EGFR-TKIs, Lung adenocarcinoma, Prognosis

## Abstract

**Background:**

Although lung adenocarcinoma with activating epidermal growth factor receptor (EGFR) mutations is common in never smokers, one-third of the patients are ever-smokers. We aimed to investigate the effect of cumulative smoking dose(CSD) on clinical outcomes, including progression-free survival (PFS) and overall survival (OS), in patients with EGFR-mutated lung adenocarcinoma receiving EGFR-tyrosine kinase inhibitors (TKIs).

**Methods:**

We retrospectively analyzed 142 patients with EGFR-mutation positive advanced or recurrent lung adenocarcinoma who were administered gefitinib, erlotinib, afatinib, and osimertinib. These patients were classified based on their CSD as never smokers, light smokers (≤10 pack-years [PYs]), moderate smokers (11–30 PYs), and heavy smokers (> 30 PYs). PFS and OS were analyzed according to smoking subgroups via Kaplan-Meier curves.

**Results:**

Among the 142 patients, 91 (64.1%), 12 (8.5%), 22 (15.5%), and 17 (12%) were never, light, moderate, and heavy smokers, respectively. CSD was inversely associated with median PFS in a statistically significant dose-dependent manner (11.8 months (mo), 11.0 mo, 7.4 mo, and 3.9 mo; *p* < 0.001). Statistically significant negative association was observed between CSD and median OS (33.6 mo, 26.3 mo, 20 mo, and 8.9 mo; *p* < 0.001). In the multivariate analysis adjusted for age, sex, performance status, stage, and timing of EGFR-TKIs, CSD was an independent predictive factor for disease progression (hazard ratio [HR], 4.00; 95% confidence interval [CI], 1.95–8.23; *p* = 0.012) and OS (HR, 3.9; 95% CI, 1.84–8.28; *p* < 0.001).

**Conclusion:**

CSD is an important predictive and prognostic factor in patients with EGFR-mutated lung adenocarcinoma, and associated smoking-related gene signatures might affect the outcomes.

**Electronic supplementary material:**

The online version of this article (10.1186/s12885-018-4691-0) contains supplementary material, which is available to authorized users.

## Background

Epidermal growth factor receptor-tyrosine kinase inhibitor (EGFR-TKI) treatment has become a standard therapy for patients with advanced lung adenocarcinoma harboring activating EGFR mutations. [1–3] Several clinical trials reported a favorable clinical response of patients with common sensitive mutations, such as frame deletions in exon 19 (19del) or point mutations in exon 21 at position 858 (21L858R), to EGFR-TKI therapy. [3–5] However, most patients experience disease progression after approximately 10–12 months of treatment, and primary resistance to EGFR-TKIs has been observed in a certain subpopulation. [3] Various mechanisms have been suggested in primary resistance to EGFR-TKIs, including smoking-related TKI resistance. [6–8] Although activating EGFR mutations are common among patients with non-small-cell lung cancer who were never smokers, approximately 30% of patients with EGFR mutations had a history of tobacco use. [9, 10]

Cigarette smoking is an independent negative prognostic factor in patients with advanced lung cancer. [4, 11, 12] Previous studies classified smoking histories into never smokers, ex-smokers, and current smokers. [11–15] The clinical outcomes and response of patients administered with EGFR-TKIs were investigated based on those categories. [16] We realized that the simple categorization of smoking history, such as never, ex-, or current smokers, is inadequate to predict the prognosis of patients with activating EGFR mutation adenocarcinoma. The effect of CSD on PFS and overall survival (OS) in patients with EGFR-mutated lung adenocarcinoma is yet to be clarified, and only few studies have directly focused on the relationship between the efficacy of EGFR-TKI and CSD in patients with EGFR-mutated lung adenocarcinoma.

In this retrospective study, we reviewed the medical records, including the smoking history of patients with positive EGFR mutations who were treated with EGFR-TKIs. We investigated whether CSD was an independent factor affecting the PFS, OS, and efficacy of EGFR-TKI in patients with EGFR mutation-positive adenocarcinoma.

## Methods

### Study population

This retrospective analysis included patients with advanced or recurrent lung adenocarcinoma harboring the EGFR exon 19del or exon 21 L858R point mutation who were administered EGFR-TKI therapy (gefitinib, erlotinib, afatinib, or osimertinib) from January 2006 to November 2016. Patients who had concomitant cancer in another organ were excluded. A total of 142 patients diagnosed with advanced-stage EGFR-mutated lung adenocarcinoma in our hospital were analyzed. Data on clinical factors, such as age, sex, smoking history, type of EGFR mutation, histopathology, clinical stage, metastatic sites, pulmonary surgical history, performance status, and treatment timing with EGFR-TKIs, were collected and analyzed.

Smoking history and dose were collected from all patients during first diagnosis. Patients were asked the number of cigarettes smoked per day (average), the number of years of smoking, and the age they started and quit smoking. Based on their CSD, the patients were classified into 4 groups as never smokers, light smokers (≤10 pack-years [PY]), moderate smokers (11–30 PY), and heavy smokers (> 30 PY). We compared the PFS and OS based on the 4 smoking subgroups. PY was calculated as the average number of cigarettes per day/20 × years of smoking.

### EGFR mutation analysis and histopathology evaluation

The samples for genetic analysis were obtained from cytology or tissue specimens before front chemotherapy. In all the cytologic and histologic samples, target tumor rich areas were marked by microscopic examination of pathologists. The tumor cells were scraped from the archived slides with 26-guage needle after the coverglass and xylene were removed. The DNA was extracted from the tumor cells. Nucleotide sequencing of the kinase domain of EGFR gene (exons 18–21) was performed by pyrosequencing method. Pyrosequencing reactions were performed according to the manufacturer’s instructions using the PSQTM96 sample preparation kit (Qiagen, UK), which contained the enzyme, substrate, and nucleotides. Nucleotide sequencing was analyzed by PyroMark ID System and SNP reagent kit (both purchased from Biotage, Uppsala, Sweden). [17]

The histopathology evaluation was performed according to the new 2015 IASLC/ATS/ERS ADC classification. We classified tumor tissues to three grades, low grade (well differentiated), intermediate grade (moderate differentiated), and high grade (poor differentiated) adenocarcinoma. Lepidic type adenocarcinoma was classified to the low grade, acinar and papillary types were classified to the intermediate grade, and micropapillary and solid types were classified to the high grade. [18]

### Evaluation of clinical outcomes

In this study, all patients received gefitinib, erlotinib, afatinib, or osimertinib treatment for recurrent or advanced lung adenocarcinoma. Patients received EGFR-TKIs until disease progression, occurrence of unacceptable toxicity, refusal of treatment by the patient, or death.

The efficacy of EGFR-TKI was evaluated by assessing tumor response every 2 months via computed tomography (CT) scan, and the highest response was recorded. The clinical responses were classified as complete response (CR), partial response (PR), stable disease (SD), or progressive disease using the Response Evaluation Criteria in Solid Tumor (RECIST version 1.1). We also examined the disease control rate (DCR) and objective response rate (ORR). Disease control was defined as the best tumor response of CR, PR, or SD that was confirmed and sustained for 8 weeks or longer, while ORR was defined as the proportion of patients with CR and PR.

PFS among patients administered EGFR-TKIs was calculated from the start of therapy to the date of clinical or radiologic progression as determined via CT imaging using RECIST criteria. While, OS was calculated from the time of diagnosis or the date of recurrence after surgical resection to the date of death from the disease or last follow-up.

### Ethics statements

The study protocol was approved by the Konkuk Medical University Hospital Institutional Review Board (Approval number: KUH1010901), and the need for written informed consent from the participants was waived due to the retrospective nature of this study.

### Statistical analyses

Pearson chi-square and Fisher’s exact tests were used to analyze categorical variables to compare baseline characteristics among the smoking subgroups. PFS and OS were estimated using the Kaplan-Meier method. The differences among the CSD subgroups were compared by using the log-rank test. Cox proportional hazard regression analysis was used to identify the effect of CSD on PFS and OS. After univariate analyses, all variables were included to create a maximal model of multivariate analysis to assess the independent effect of CSD. The ORR and DCR among smoking subgroups were evaluated via Pearson’s chi-square test or Fisher’s exact test. Parametric variables were compared using the independent sample *t*-test, while the relationship between non-parametric variables was assessed using the chi-square test. The linear regression method was used to predict the PFS and survival time based on CSD. A *p* value of < 0.05 was considered statistically significant. All statistical analyses were performed using the statistical software SPSS version 23.0 (SPSS, Inc.; Chicago, IL, USA).

## Results

### Patient characteristics

A total of 142 patients with advanced or recurrent lung adenocarcinoma with susceptible EGFR mutation treated at Konkuk University Medical Center, Seoul, Korea were retrospectively reviewed between January 2006 and November 2016. The median age of all patients was 65 years (range, 54.3–75.7 years). Of the 142 patients, 64.8% were women, and 123 (86.6%) had good Eastern Cooperative Oncology Group (ECOG) performance status of 0–1. A total of 91 patients (64.1%) were never smokers, and 51 patients (35.9%) were ever-smokers. Of the 51 ever-smokers, 28 were ex-smokers, and 23 were current smokers. Based on the CSD, 12 patients were light smokers with 0–10 PYs, 22 patients were moderate smokers with 11–30 PY, and 17 patients were heavy smokers with more than 30 PY. The majority of never smokers were women, whereas most ever-smokers were men. The proportion of male smokers increased as the CSD increased as shown in Table 1 (*p* = 0.001). The clinical characteristics of the patients stratified by CSD were similar and were distributed equally except for sex (Table 1). The majority of the patients (*n* = 107 [75.4%]) received gefitinib as the EGFR-TKI, and approximately 59.2% were treated with EGFR-TKIs as first-line treatment. The median follow-up period was 19.8 months. We evaluated the histopathology of the 94 patients of the total 143 patients by 2015 WHO classification of lung tumors but could not classify the pathologic type of other patients because their data were from bronchial washing (*n* = 10), pleural fluid (*n* = 19) and metastatic tissue (*n* = 14). We observed mainly the acinar type (54.3%) in EGFR mutation-positive adenocarcinoma and the proportion of solid type increased in the ever- smoker. (Additional file 1: Table S1).

**Table 1:** Patient baseline characteristics

N (%)	Total	Never smoker	0< PY^a^ ≤10	10<PY^a^ ≤30	> 30^a^ PY	*p*-value
	142	91(64.1)	12(8.5)	22(15.5)	17(11.9)	
Age	65(54.5–75.7)					
< 60 years	49(34.5)	33(36.3)	3(25)	8(36.4)	5(29.4)	0.842
≥ 60 years	94(65.5)	58(63.7)	9(75)	14(63.6)	12(70.6)	
Sex						
male	50(35.2)	10(11)	8(66.7)	18(81.8)	14(82.4)	0.001
Female	92(64.8)	81(89)	4(33.3)	4(18.2)	3(17.6)	
ECOG						
0–1	123(86.6)	81(89)	11(91.7)	19(86.4)	12(70.6)	0.23
≥ 2	19(13.4)	10(11)	1(8.3)	3(13.6)	5(29.4)	
Stage^b^						
recurrent	15(10.5)	12(13.2)	19(8.3)	1(4.5)	1(5.9)	0.33
IIIA	7(4.9)	5(5.5)	1(8.3)	1(4.5)	0(0)	
IIIB	12(8.5)	8 (8.8)	0(0)	0(0)	4(23.5)	
IV	108(76.1)	66(72.5)	10(83.3)	20(90.9)	12(70.6)	
M1a	40(37)	24(36.4)	3(30)	7(35)	6(50.0)	0.77
M1b	68(63)	42(63.6)	7(70)	13(65)	6(50.0)	
Time of EGFR-TKI treatment						
First line	84(59.2)	60(65.9)	6(50)	11(50)	7(41.2)	0.16
Second or higher line	58(40.8)	31(34.1)	6(50)	11(50)	10(58.8)	
EGFR mutation						
19 del	91(64.1)	59(64.8)	10(83.3)	13(59.1)	9(52.9)	0.38
21 L858R	51(35.9)	32(35.2)	2(16.7)	9(40.9)	8(47.1)	
Type of EGFR TKI						
Gefitinib	107(75.4)	71(78)	6(50.0)	17(77.3)	13(76.5)	0.53
Erlotinib	17(12)	9(9.9)	2(16.7)	3(13.6)	3(17.6)	
Afatinib	14(9.9)	9(9.9)	3(21.4)	1(4.5)	1(5.9)	
Osimertinib	4(2.7)	2(2.2)	1(8.3)	1(4.5)	0(0.0)	
Brain metastasis						
Yes	42(29.6)	26(28.6)	5(41.1)	9(40.9)	2(11.8)	0.19

Abbreviation: *PY* pack-years, *ECOG* Eastern Cooperative Oncology Group, *EGFR* epidermal growth factor receptor, *TKI* tyrosine kinase inhibitor

^a^Of the 51 ever-smokers, 28 were ex-smokers, and 23 were current smokers

^b^Clinical stage at the time of initial diagnosis was determined according to the American Joint Committee on Cancer (7th edition)

### Response to EGFR-TKIs according to cumulative smoking dose

We analyzed the association between patient response to EGFR-TKIs and CSD. The ORR and DCR were compared among the smoking subgroups. The ORR to EGFR TKIs was decreased significantly as CSD increased (*p* = 0.003). However, the DCR of never smokers, light smokers, moderate smokers, and heavy smokers was similar at approximately 80 ~ 90% regardless of the CSD (Table 2). No significant difference in DCR was observed among the smoking subgroups (*p* = 0.39).

**Table 2:** Comparison of treatment response rate according to cumulative smoking dose

*N* = 135, N (%)	Never smoker (*n* = 91)	0 < PY ≤ 10 (*n* = 12)	10 < PY ≤ 30 (*n* = 22)	> 30PY (*n* = 17)	p
ORR (%)	66 (72.5%)	11 (91.7%)	12 (54.5%)	6 (35.3%)	0.003
DCR (%)	83 (91.2%)	12 (100%)	18 (81.8%)	15 (88.2%)	0.39
CR, N (%)	1 (1.1%)	0 (0%)	0 (0%)	0 (0%)	
PR, N (%)	65 (71.4%)	11 (91.7%)	12 (54.5%)	6 (35.3%)	
SD, N (%)	17 (18.7%)	1 (8.3%)	6 (27.3%)	9 (52.9%)	
PD, N (%)	1 (1.2%)	0 (0%)	4 (18.2%)	2 (11.8%)	
unevaluable	7 (7.7%)	0 (0%)	0 (0%)	0 (0%)	

Abbreviation: *PY* pack-years, *ORR* objective response rate, *DCR* disease control rate, *CR* complete response, *PR* partial response, *SD* stable disease, *PD* progressive disease, ORR(CR + PR), DCR(CR + PR + SD)

### Progression-free survival and overall survival according to cumulative smoking dose

**Fig. 1:**
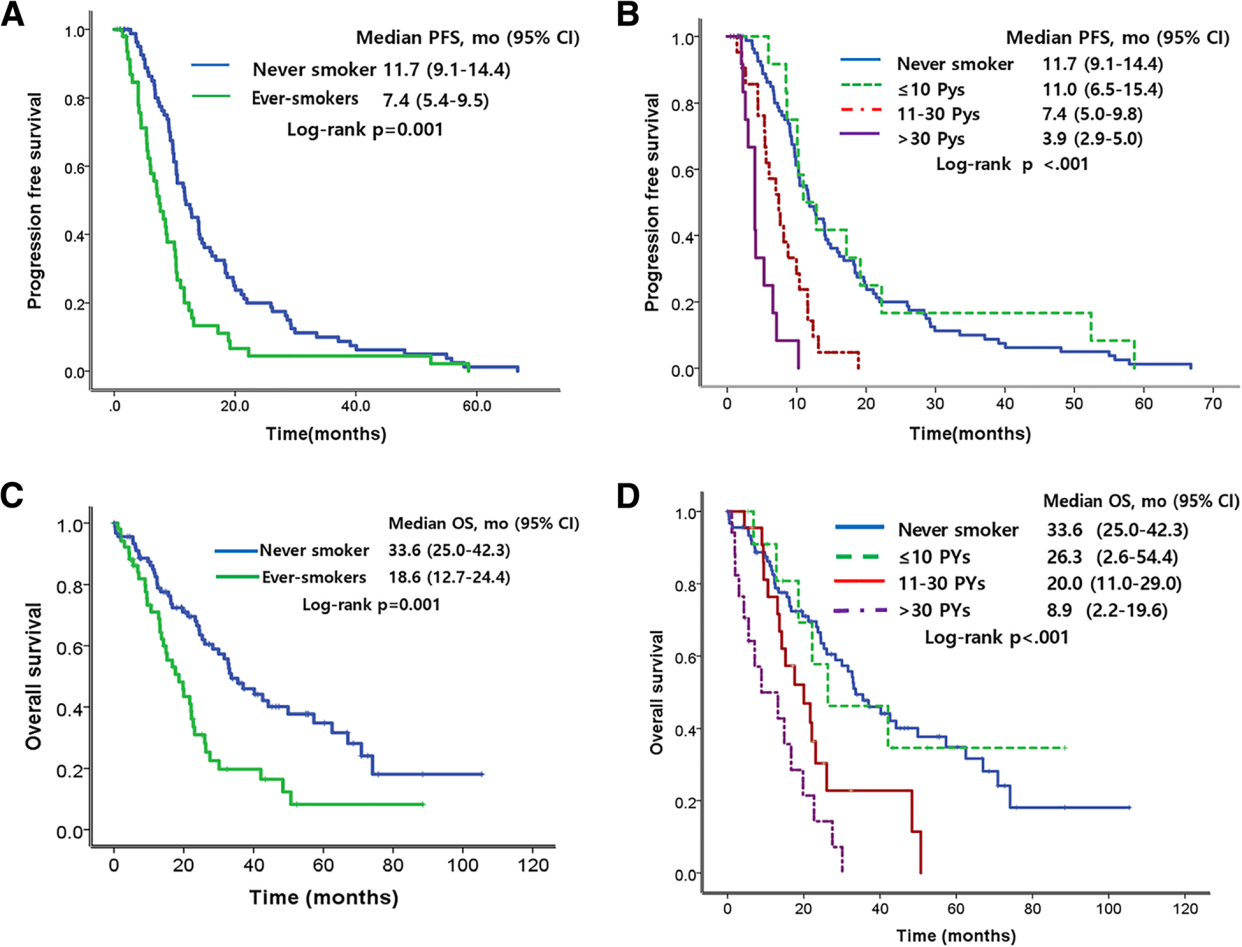
Comparisons of PFS and OS according to cumulative smoking dose in patients receiving EGFR-TKIs. Comparison of PFS by (**a**) smoking history, and (**b**) cumulative smoking dose. Comparison of OS by (**c**) smoking history and (**d**) cumulative smoking dose

The median PFS among the 142 patients was 10.3 months (95% CI: 9.6–10.9). The median PFS of the never smokers and ever-smokers was 11.7 months, 7.4 months respectively (*p* = 0.001; Fig. 1a). This is consistent with previous studies that reported a shorter PFS for smokers. To investigate how the amount of smoking is related with PFS, we subdivided CSD into four classes. The median PFS of the never smokers, light smokers, moderate smokers, and heavy smokers was 11.7 months, 11.0 months, 7.4 months, and 3.9 months, respectively (*p* < 0.001; Fig. 1b). The PFS of the light smokers group was similar to that of the never smokers group (11.0 months [95% CI: 6.5–15.3] vs 11.7 months [95% CI: 9.1–14.3]; log-rank *p* = 0.63). Smokers with high CSD had significantly short PFS (log-rank p < 0.001). CSD was inversely associated with median PFS in a dose-dependent manner with statistical significance (Fig. 1b).

In the 142 patients, the median OS was 26 months (95% CI: 20.2–31.8). The median OS of the never smokers and ever smokers was 33.6 months, 18.6 months respectively (*p* = 0.001; Fig. 1c). The median OS of never smokers, light smokers, moderate smokers, and heavy smokers was 33.6 months (95% CI: 24.9–42.3), 26.3 months, (95% CI: 2.6–54.4), 20.0 months (95% CI: 11.0–29.0), and 8.9 months (95% CI: 2.2–19.6), respectively (*p* < 0.001; Fig. 1d). The OS was similar in the light smoker group with less than 10 PYs and in the never smoker group. OS decreased as the CSD increased. (log-rank *p* < 0.001).

### Multivariate analysis of survival outcome on EGFR-TKIs therapy

In univariate analysis of PFS, moderate and heavy smokers with a cumulative smoking history of more than 10 PY had significantly shorter PFS than that of never or light smokers. Additionally, Cox proportional hazard model showed that the advanced stage (*p* = 0.02) and brain metastasis (*p* = 0.001) also significantly poor prognostic factors in the PFS of our patients (Table 3). In univariate analysis of OS, moderate and heavy smokers with a cumulative smoking history of more than 10 PY also had significantly higher hazard ratio (HR) than that of never or light smokers. In the other factors, sex, performance status, stage, brain metastasis, and lines of EGFR-TKIs significantly affected the OS in univariate analysis (Table 3). Results of the univariate Cox regression analysis indicated that PFS and OS were negatively correlated with CSD of more than 10 PYs. However, we could not find significant difference depending on histologic type in comparison of PFS and OS (Table 3 and Additional file 2: Figure S1).

**Table 3:** Univariate analysis via Cox- regression model of the influence of clinicopathologic variables on survival outcomes in patients with EGFR mutation receiving EGFR-TKIs

variable	category	PFS		OS	
		HR (95% CI)	*p*-value	HR (95% CI)	*p*-value
Smoking history	ever-smokers vs. never smoker	1.9(1.31–2.76)	0.001	2.206(1.43–3.40)	< 0.001
Smoking dosage	never smoker (PY = 0, Ref.)	1		1	
	0 < PY ≤10	0.84(0.45–1.55)	0.08	0.96(0.41–2.25)	0.93
	11~ 30 PY	2.95(1.76–4.94)	< 0.001	2.29(1.29–4.05)	0.004
	> 30 PY	8.33(4.18–16.75)	< 0.001	4.91(2.66–9.06)	< 0.001
	11~ 30 PY vs. > 30 PY	2.97(1.35–6.56)	0.007	2.22(1.08–4.57)	0.03
Age	> 60 vs. ≤ 60 years	1.05(0.72–1.53)	0.78	0.99(0.64–1.53)	0.98
Sex	Male vs. female	1.39(0.78–1.62)	0.084	1.91(1.238–2.947)	0.003
ECOG PS	≥ 2 vs 0–1	1.76(0.99–3.12)	0.05	3.12(1.73–5.62)	< 0.001
Stage	IV vs. recurrent or III	1.23(1.03–1.46)	0.02	1.4(1.1–1.9)	0.02
Type of EGFR mutation	19 del vs. 21 L858R	1.2(0.81–1.7)	0.36	0.66(0.38–1.15)	0.15
Brain metastasis		1.9(1.3–2.9)	0.001	2.54(1.46–4.41)	0.001
Line of targeted therapy	≥2nd line vs. 1st	1.15(0.80–1.64)	0.439	1.92(1.25–2.93)	< 0.001
Histopathology	High vs. low to moderate grade adenocarcinoma	1.17(0.81–1.69)	0.39	0.91(0.51–1.65)	0.78
Type of EGFR-TKI		0.95(0.75–1.18)	0.63	1.2 (0.53–2.69)	0.66

Abbreviation: *HR* hazard ratio, *CI* confidence interval, *ECOG PS* Eastern Cooperative Oncology Group performance status

*EGFR* epidermal growth factor receptor

Hazard ratios and *p* values are adjusted for patients’ age (≥60 years vs. < 60 years), sex (female vs. male), ECOG status (PS status 0–1 vs. PS status 2–4), initial tumor stage (stage IV vs. III or recurrent), and line of EGFR-TKI (≥2nd vs. 1st line [Ref.]) in Cox-proportional hazard model

In the multivariate analysis adjusted for age, sex, ECOG status, initial stage, and timing of targeted therapy, CSD of more than 10 PYs was a significant and independent predictive factor for disease progression after EGFR-TKIs treatment (moderate smokers [11–30 PYs]: HR, 4.00; 95% CI, 1.95–8.23 and heavy smokers [> 30 PYs]: HR, 16.2; 95% CI, 6.37–61.6; *p* < 0.001, Table 4). In these comparisons, the HR of light smokers with less than 10 PY was not different with that of never smokers (*p* = 0.49). The multivariate analysis of OS also showed that CSD of more than 30 PYs was a negative predictive factor of OS (HR 3.98; 95% CI: 1.84–8.28; p < 0.001) (Table 3). A CSD of ≥30 PY independently predicted poor PFS and OS for EGFR-TKIs treated patients with activating EGFR mutations.

**Table 4:** Multivariate analysis of the predictive value of cumulative smoking dose on survival outcomes in patients with EGFR mutation receiving EGFR-TKIs

variable	category	PFS to EGFR-TKI		*p*-value	OS	95% CI	*p*-value
		HR	95% CI		HR		
Smoking history	ever- smokers vs never smoker	1.89	1.27–2.81	0.002	1.52	0.87–2.64	0.13
Smoking dosage	never smoker	1.00			1.00		
	≤10 PY	0.81	0.43–1.50	0.49	0.83	0.32–2.14	0.68
	11~ 30 PY	4.00	1.95–8.23	< 0.001	1.52	0.79–2.96	0.21
	> 30 PY	16.2	6.37–61.6	< 0.001	3.98	1.84–8.28	< 0.001

Adjusted by Age, PS, Sex, clinical stage and time of targeted therapy

Abbreviations: *PFS* progression-free survival, *OS* overall survival, *Ref*. reference, *EGFR* epidermal growth factor receptor, *TKI* tyrosine kinase inhibitor, *PS* performance status, *HR* hazard ratio, *95% CI* 95% confidence interval, *PY* pack-years

### Simple linear regression to predict survival outcomes based on CSD in smokers with EGFR positive adenocarcinoma

**Fig. 2:**
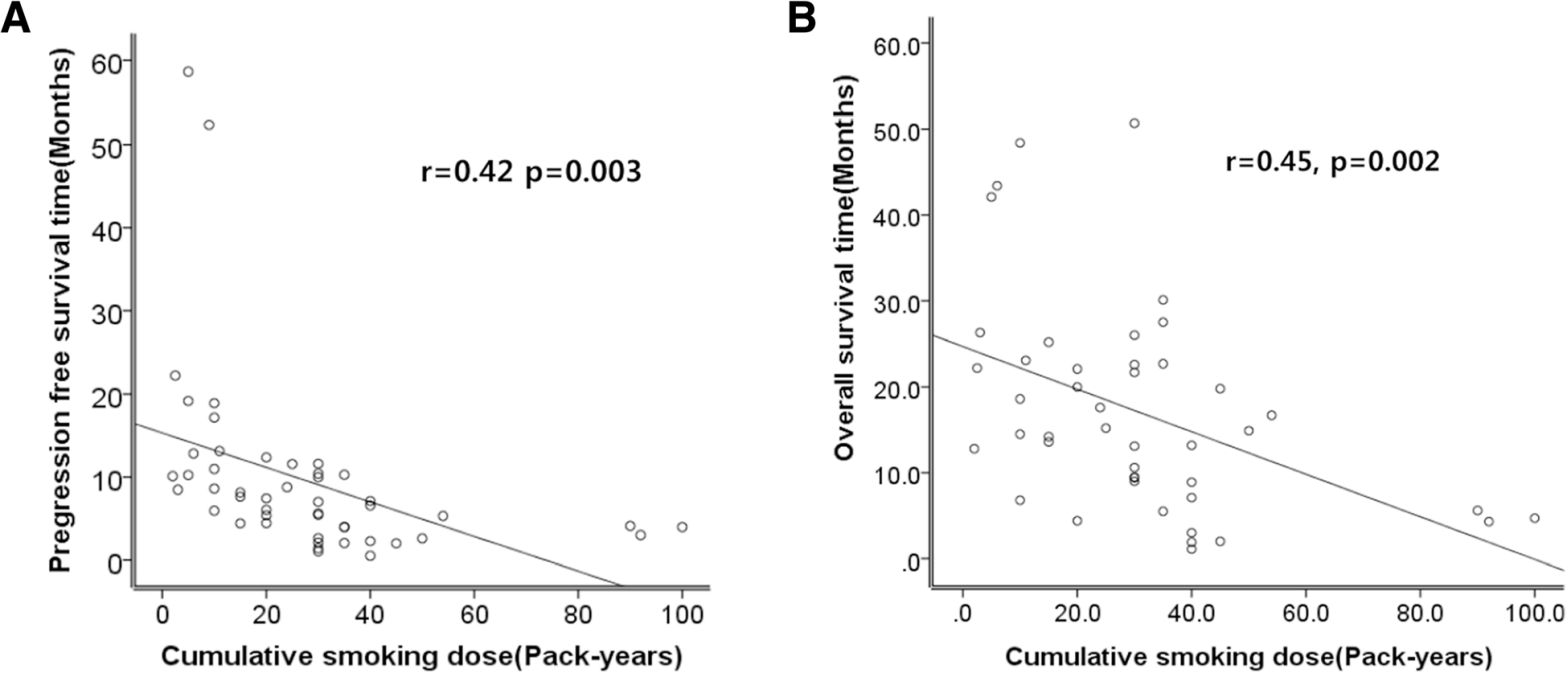
Relationship between clinical outcomes, (**a**) PFS and (**b**) OS and cumulative smoking dose. The PFS or OS was significantly inversely associated with the CSD

We investigated the correlation between CSD and disease progression or OS via simple linear regression analysis. The progression-free survival time of the 51 patients with disease progression after EGFR-TKIs was plotted based on cumulative smoking dose (Fig. 2a). The overall survival period of 44 patients who died among smoker plotted based on CSD (Fig. 2b). We excluded never smokers in this analysis because their PFS and OS were commonly long and varied due to primary resistance. Even in this method, we found the inverse correlation between PFS or OS and CSD (PFS: *r* = 0.42, *p* = 0.003; OS *r* ^=^ 0.45, *p* = 0.002). The β coefficient value of PFS was − 0.20±0.06 and that of OS was − 0.25±0.08. CSD might predict the approximate PFS and OS time in the patient with EGFR mutation -positive adenocarcinoma through this graph.

## Discussion

In this study, patients diagnosed with advanced lung adenocarcinoma harboring susceptible EGFR mutations and treated with EGFR-TKI were grouped based on CSD, and the PFS and OS were compared among the smoking subgroups. The results showed that patients with high CSD tended to have poorer PFS and OS than those with low CSD. Previous studies have showed that CSD of more than 30 PYs affected the PFS. [19] In our study, we observed that moderate CSD (11–30 PYs) is also an independent negative predictor of PFS in addition to CSD more than 30 PYs. Heavy smokers with 30 PYs treated with EGFR-TKIs had shorter PFS than those treated with conservative chemotherapy (5–6 mo). [2–4, 20] The PFS and OS of light smokers (≤10 PYs) were similar with that of never smokers (PFS: *p* = 0.81; OS: *p* = 0.92).

CSD is also an important independent negative predictive factor of EGFR-TKI treatment efficacy. Although that the DCR of TKI is over 80–90% irrespective of smoking subgroups., the ORR was decreased as the CSD increased (Table 2, *p* = 0.003). The fact indicated EGFR-TKIs suppress tumor growth through its tyrosine kinase inhibitor and is highly effective as evidenced by the high DCR of over 80–90%. However, the duration of the drugs was shorter based on CSD as evidenced by the shorter PFS of moderate smokers (median PFS: 7.4 months) and heavy smokers (median PFS: 3.9 months) than that of never or light smokers (median: 11.8 months and 11.0 months) (*p* < 0.001). EGFR-TKIs can initially prevent disease progression, but the maintenance of a disease-free period would be short if the CSD is high. CSD might predict the approximate PFS or OS through linear regression graph shown Fig 2. Our study showed that in addition to smoking status, CSD is an important independent negative predictive factor of EGFR-TKI treatment outcome and efficacy in patients with lung adenocarcinoma with activating EGFR mutation.

We compared the PFS and OS according to histopathology subtypes, but significant difference was not found (Additional file 2: Figure S1). Some of non-smoker patients in the solid type showed longer PFS than smoker patients in the acinar type. However, the ratio of smokers in the solid type tends to be higher than those in the other types, though statistical difference is not significant (Additional file 1: Table S1, *p* = 0.08). The previous researches reported the poor prognosis of the solid type. The poor prognosis of solid type in previous researches might be due to the smoking not pathologic types, because many solid types are usually found in smokers. The prognosis was more affected by molecular testing rather than the histology subtype in the patients with EGFR mutation-positive lung adenocarcinoma. Our results was different from the previous studies since we focused only on the patients with only EGFR mutation-positive adenocarcinoma. [21, 22] Also, the difference may be caused by the small biopsies, the heterogeneous mixture of lung tissue, and the limited number of patients. [18] More investigation is needed to address the relevance of histological grading and prognosis of patients treated with EGFR-TKIs.

Table 3 shows that CSD with more than 10 PYs is associated with short PFS and OS in univariate analysis of Kaplan-Meier curve. Advanced clinical stage and brain metastasis was associated with short PFS as in the previous report. [23, 24] Patients with exon-19 deletion tended to show more favorable outcomes than those with exon-21 L858R in previous reports. [16, 25, 26] By contrast, no significant difference in PFS and OS was observed in our study. In recent researches, patients treated with first-line TKI therapy showed longer PFS and OS than those treated with TKI as second-line therapy. [24, 27, 28] In our study, 70.6% of the patients with 21 L 858R were treated with EGFR-TKIs as the first-line therapy, while 47.3% of patients with 19 del were treated with it as the first-line therapy. Relatively more EGFR-TKIs as a first line were administered in patients with 21del mutation. This contributed to the longer PFS and OS in patients with exon 21 mutation than in patients with 19 del. Univariate analysis of OS showed that male sex, poor performance status, and high line of TKI therapy are associated with short OS. The advantage of our study was that more than half (59.2%) of the patients was treated with EGFR-TKIs as first-line therapy, while most (81.6%) patients in the previous study were treated with EGFR-TKIs as second-line treatment. [19] The PFS and OS of the patients treated with second line EGFR-TKI followed by previous chemotherapy would be short because chemotherapy may induce more genetic alterations in cancer lesion, decreased TKI sensitivity and decrease the patient’s performance status. [29] Therefore, the previous study may not show the difference among smoking subgroups and the results cannot be applied to the current post-TKI era when EGFR-TKIs is primarily used as first-line therapy.

The poor survival outcome and the low response rate to the EGFR-TKI in smokers with activating EGFR mutations might be attributed to several reasons. EGFR mutation might be among the carcinogenic mechanisms in the development of cancer in smokers with activating EGFR mutation. Other genetic alterations might also be associated with smoking, such as point mutations of p53, *K*-*ras* gene activation, high number of single nucleotide variants and structural mutation [6, 7]. Moreover, downstream activation of AKT, ERK(extracellular-regulated kinase) pathway [8] and Src signaling pathways via nicotine exposure might contribute to tumor growth. [30] These alterations might mediate the resistance to EGFR TKIs [6, 7, 31] indicating that EGFR-TKIs alone cannot completely block other downstream carcinogenic pathways induced by cigarette smoking. Preclinical studies have shown that cigarette smoking activates the EGFR pathway and induces conformational change of EGFR receptors, resulting in downstream activation via c-Src and Cav-1 binding. [32] Another mechanisms for the development of cancer have also been suggested, including the activation of the nicotinic acetylcholine receptor [33] and epithelial to mesenchymal transition(EMT). [34] Smoking also affect the pharmacokinetic and pharmacodynamic properties of EGFR-TKIs. Long-term repeated exposure to nicotine during tumor development would also induce more genetic alterations and posttranslational conformational change of EGFR receptor, causing TKI resistance. Several mechanisms may be related to primary resistance to EGFR-TKIs, such as low EGFR gene copy number [35, 36] and intra-tumor genetic heterogenicity. [37, 38] Although our data on about smoking status during treatment were not obtained and only CSD at diagnosis was acquired, the effect of CDS predominantly affects the prognosis of smoker in whole time. Further preclinical studies should investigate whether the conformational change and secondary pathway phosphorylation of EGFR receptor by past nicotine exposure influenced consistently the prognosis.

This survival analysis had several limitations. First, this was a retrospective study with a limited size of patients in Asian ethnicity from one medical center. Our results should be confirmed by further similar research based on various ethnicity. Second, EGFR-TKIs were not randomly assigned to the smoking subgroups, but this could be compensated by the equal proportion of types of EGFR-TKIs among the smoking subgroups. Fortunately, the prescriptions of the four EGFR-TKIs were balanced among the smoking subgroups. Third, data on smoking history was only collected during first diagnosis, and the smoking status during treatment was not followed-up despite that current smoking might influence the efficacy of EGFR-TKIs. The mechanisms of resistance to EGFR-TKIs include substantial primary resistance and smoking-related resistance. For smoking patients with EGFR mutation, prospective collection of tissue for molecular analysis or liquid biopsy [39] is essential for extensive molecular and genetic analyses, including next-generation sequencing (NGS). The introduction of NGS and liquid biopsy into cancer genetic research enables the measurement of tumor mutation burden and smoking-related gene signature. [40] The heavy smokers with activating EGFR mutation have high mutation burden, [6] and immunotherapy would be applicable for them. [41] The survival outcomes in patients treated with second-line immunotherapy vs second-line chemotherapy after failure of EGFR-TKIs treatment should also be analyzed through further clinical studies.

Lastly, we should focus on the poorer survival outcomes of heavy smokers with 30 PYs treated with EGFR-TKI (median PFS: 3.9 months; median OS: 8.9 months) than those treated with standard chemotherapy (median PFS: 5–6 months). [4, 20] Such results suggests that other treatment options, such as chemotherapy or a combination of Src inhibitors, in addition to EGFR-TKIs should be considered in treating heavy smokers. Some clinical trials showed that the concurrent combination of chemotherapy and gefitinib induced high ORR, long median PFS, and acceptable toxicity. [42–44] Combining EGFR-TKIs with chemotherapy should be considered in the treatment of patients with CSD of more than 30 PYs because the efficacy of EGFR-TKIs is lower than standard chemotherapy in heavy smokers. The Src pathway mediates cigarette smoke-induced resistance, [45] and Src inhibitors such as Dasatinib block downstream signaling pathways, resulting in both suppression of cell growth and induction of apoptosis. [46] N-acetylcysteine (NAC) also decreases Src phosphorylation, abrogating changes of EMT. [32] Therefore, the combination of dasatinib or NAC with EGFR-TKIs can be considered as a new treatment option for heavy smokers with activating EGFR mutation to prolong their PFS and enhance drug efficacy.

## Conclusion

Our study found that CSD is an important independent negative predictive and prognostic factor to EGFR-TKI treatment outcome in patients with EGFR-mutated lung adenocarcinoma. The detailed smoking history including CSD should be collected at the initial diagnostic work-up stage. It is highly plausible that EGFR-mutated advanced lung adenocarcinoma developed in smokers is not only attributed to the oncogenic EGFR mutation but also other smoking-related co-occurring genetic alterations. To find out concomitant genetic alterations, the next generation sequencing should be encouraged to perform in addition to the PCR-based EGFR mutation detection. Additionally, the multiple combination therapeutic strategy with EGFR-TKIs should be considered based on concurrent genetic signatures.

## Additional files

Additional file 1 **: Table S1.** Classification of histopathologic subtype according to smoking status in EGFR-positive lung adenocarcinoma. The proportion of solid type in the ever- smoker is higher than that in never smoker. (DOCX 17 kb)

Additional file 2 **: Figure S1.** Comparisons of (A)PFS and (B) OS according to the tumor grade in patients receiving EGFR-TKIs. We could not find significant difference depending on histologic types in comparison of PFS and OS. Tumor was graded as follows. The low-grade group includes lepidic type, the intermediate-group includes acinar and papillary types, and the high-grade group includes micropapillary and solid type. (TIF 3615 kb)

## Abbreviations

CI: Confidence interval
CR: Complete response
CSD: Cumulative smoking dose
CT: Computed tomography
DCR: Disease control rate
ECOG: Eastern Cooperative Oncology Group
EGFR: Epidermal growth factor
EGFR-TKI: Epidermal growth factor receptor-tyrosine kinase inhibitors
EMT: Epithelial to mesenchymal transition
HR: Hazard ratio
mo: Months
NAC: N-acetylcysteine
NGS: Next-generation sequencing
ORR: Objective response rate
OS: Overall survival
PFS: Progression-free survival
PR: Partial response
PS: Performance status
PY: Pack-years
RECIST: Response Evaluation Criteria in Solid Tumor
SD: Stable disease
