# Supplementary material for: Cumulative smoking dose affects the clinical outcomes of EGFR-mutated lung adenocarcinoma patients treated with EGFR-TKIs: a retrospective study
Source: BMC Cancer. 2018 Jul 28;18:768. doi: 10.1186/s12885-018-4691-0 (PMC6064083; doi:10.1186/s12885-018-4691-0)
Supplement: Supplementary file 1 — : Table S1. Classification of histopathologic subtype according to smoking status in EGFR-positive lung adenocarcinoma. The proportion of solid type in the ever- smoker is higher than that in never smoker. (DOCX 17 kb) [file 12885_2018_4691_MOESM1_ESM.docx]

**Table S1. Classification of histopathologic subtype according to smoking status in EGFR-positive lung adenocarcinoma.**

| **Histopathology** | **N (%)** | **Never smoker** | **Ever-smoker** | |  | |
| --- | --- | --- | --- | --- | --- | --- |
| Lepidic type | 15(16.0) | 12(18.2) | 3(10.7) |  | |  |
| Acinar type | 51(54.3) | 39(59.1) | 12(42.8) |  | |  |
| Papillary type | 4 (4.3) | 3(4.5) | 1(3.6) |  | |  |
| Micropapillary type | 11(11.7) | 7(10.6) | 4(14.3) |  | |  |
| Solid type | 13(13.8) | 5(7.6) | 8(28.6) |  | |  |
| **Total** | 94 | 66 | 28 |  | |  |
