# Supplementary figures and images for: Cumulative smoking dose affects the clinical outcomes of EGFR-mutated lung adenocarcinoma patients treated with EGFR-TKIs: a retrospective study
Source: BMC Cancer. 2018 Jul 28;18:768. doi: 10.1186/s12885-018-4691-0 (PMC6064083; doi:10.1186/s12885-018-4691-0)

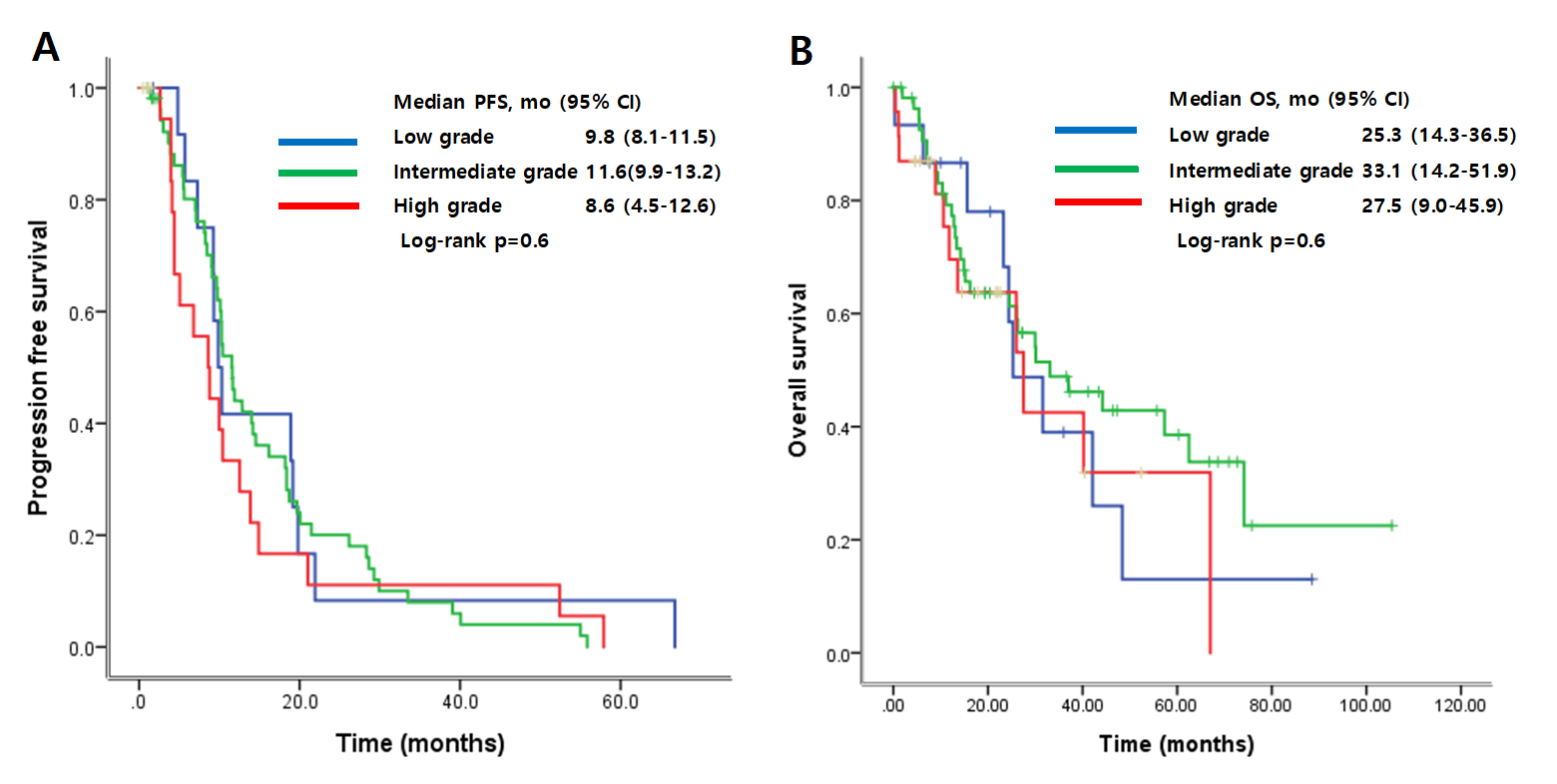

Supplement: Supplementary file 2 — : Figure S1. Comparisons of (A)PFS and (B) OS according to the tumor grade in patients receiving EGFR-TKIs. We could not find significant difference depending on histologic types in comparison of PFS and OS. Tumor was graded as follows. The low-grade group includes lepidic type, the intermediate-group includes acinar and papillary types, and the high-grade group includes micropapillary and solid type. (TIF 3615 kb) [file 12885_2018_4691_MOESM2_ESM.tif]
